# Supplementary material for: What enabled the successful implementation of a quality certification initiative in Bhavnagar, Gujarat? A policy analysis case study
Source: PLOS Glob Public Health. 2025 Jan 21;5(1):e0004180. doi: 10.1371/journal.pgph.0004180 (PMC11750092; doi:10.1371/journal.pgph.0004180)
Supplement: S1 File — (DOCX) [file pgph.0004180.s001.docx]

**TOOLS USED FOR DATA COLLECTION**

**INTERVIEWS ON NQAS WITH KEY INFORMANTS (POLICY LEVEL)**

| **Topic** | **Probes** |
| --- | --- |
| Rapport building | Introduction, ask something about the person, general conversation |
|  | Bhavnagar has got many of its HWCs certified successfully. Could you tell us your opinion on why this is so.   - What made things different in Bhavnagar compared to other districts - What were some of the unique mechanisms that Bhavnagar adopted   (probe on the peer mentoring mechanism)   - Did you make any tweaks to the process of NQAS certification according to the Bhavnagar reporting system? - What are key measures you take for successful certification of facilities (Dealing with external, Support of District and Taluka level officials during the assessment) |
| General questions about NQAS policy level decisions | - Could you tell us something about the NQAS certification process? - Who all are involved in this from the district administration? (how does the hierarchy look like) - Who is ultimately responsible? - Who takes day-to-day decisions and helps the facilities at the district level? - Who does the monitoring? - Who decides on the funds related matters during the NQAS certification? |
| Selection of facilities | - Any interesting things that came up in the way the facilities get selected. - Who selects the facilities for NQAS? What does this process look like? (Probes- Does it involve some informal criteria or a systematic process is followed?) - Can you tell us about internal assessment? How is this process undertaken? - What are some of the informal criteria that are used for this? - What is done if a facility comes forward and is interested in NQAS? - Who makes the decisions on which facilities must get NQAS certified first? What are these decisions based on?   (Probes- ask people to talk through the last time they selected a facility or block for NQAS certification)   - Can a facility refuse to get NQAS certified? How do you deal with hesitancy and disinterest at the facility level? - Now that more than 70% of PHCs are done with certification, what is your strategy for selection of HWCs and how do you plan to go about with other PHCs? |
| Processes | - Could you describe the entire process of certification? - Innovations made in the processes- like peer mentoring.   What innovations in the NQAS processes have helped Bhavnagar?   - Which are the challenging parts in selection of facilities or during the preparation for certification? How to do deal with such challenges? - Which are the easy parts of the process? Could you tell us some stories of the most challenging facility you had to get certify? - Can you please tell us if there is any scope for improvement of NQAS certification process? If you were given full freedom to do the NQAS certification process differently, how would you go about it?   Probes-   - Are there portions in the NQAS certification that are more useful than others? - What portions can be deleted? - What can be added to the certification process. - What part of the process should be followed irrespective of Certification? - How is the certification process different between PHC and SC? |
| Advantages and disadvantages | - What is your opinion on NQAS? Do you feel the certification of NQAS is useful to the district in any manner? How does NQAS support other health related policies in the district? - How does NQAS support HWCs? (incentives, improvement in skills, maintain of registers, cross referrals, signages, infrastructure, HR) Or Do staff find value in the NQAS process? Why and why not? - Do you think NQAS certification is a good way to improve quality of services?   -Quality of care provided to people  -in what ways does NQAS certification help improve quality?  (signages, patient safety, service delivery, skills of healthcare providers, better record keeping, navigation, availability of services)  -are there other ways to improve quality of services? |
|  | - What is your opinion on the NQAS certification policy for HWCs? - Do you think there are advantages in doing NQAS certification?   Are there any disadvantages? (increased OPD, increased hygiene and sanitation, better working conditions, increased patient satisfaction, increased availability of kits and other materials, better teamwork)   - How does it impact of quality of care provided at the facility? - Will you be interested to carry out the process if it was not mandatory by government? Why? - Are there other ways of improving quality of health facilities (that may be better than NQAS?)   Can try  What is meant by quality?  What aspects of quality can certification improve?  What aspects will it not help with? |
| Success stories and lessons | - Could you share with us your best success stories of NQAS? (Probe: What happened in these facilities that made NQAS possible?) - Could you share with us your experiences with NQAS certification of HWCs? (Ask for stories) - Could you tell us 3-4 suggestions that you have for other states that have not yet begun the NQAS certification processes. - Any other certifications happening in Gujarat? How have those contributed to successful NQAS certification? |
| Challenges | - Could you tell us some of the challenges you face at present with NQAS - Could you tell us a little about the sustainability of the improvements made, after certification? (Monthly refreshers, maintaining of registers, regular maintain of records, maintaining the placements of things according to labelling) - Could you please explain how you dear with challenges related to finances, HR or other key aspects. Others? |

**INTERVIEWS ON NQAS WITH KEY INFORMANTS AT FACILITY LEVEL**

| **Topic** | **Probes** |
| --- | --- |
| Rapport building | Introduction, ask something about the person, general conversation |
| General questions about NQAS policy level decisions | - Could you tell us something about the NQAS certification process - Who all are involved in the process of certification in your institute? - Who all are involved in this from the district administration? (how does the hierarchy look like) - Who is most crucial for undertaking NQAS certification of your facility? Who takes day-to-day decisions and helps the facilities? - Who does the monitoring? What is your role in mentoring others at your facility? - Can you tell us more about the support you have received from District administration/ other facilities/ state and others like NGOs. |
| Selection of facilities | - Who selects the facilities for NQAS? What does this process look like? (Probes- Does it involve some informal criteria or a systematic process is followed?) How did your facility get selected for NQAS? - Can you tell us about internal assessment? How is this process undertaken? What are some of the informal criteria that are used for this? - Can you or anyone at the facility refuse to get NQAS certified? - What is your role in selection of sub centers under your PHC for NQAS certification? |
| Processes | - Could you describe the entire process of certification? - What are your thoughts regarding the mentoring help provided by the district? - Which are the challenging parts? Which are the easy parts of the process? - Could you share with us your experiences with NQAS certification of HWCs? (Ask for stories) - How can we improve this process - Any other certifications happened for your facility? How has that contributed to the successful NQAS certification of your facility? - What is your opinion on the NQAS certification of the sub centers that has started in Bhavnagar? |
| Advantages and disadvantages | - In your experiences, what do HWC staff think of the process of NQAS certification? - Do you feel the certification is useful to the HWC/PHC in any manner? In what ways does it help the HWCs? Or Do staff find value in the NQAS process? Why and why not? - Experiences: Do you think NQAS certification is a good way to improve quality of services? - Probes   -in what ways does NQAS certification help improve quality?  -are there other ways to improve quality of services?  Do you think there are advantages in doing NQAS certification?  Are there any disadvantages?)  Can try: What is meant by quality?  What aspects of quality can certification improve?  What aspects will it not help with? |
| Success stories and lessons | - Could you share with us your best success stories of NQAS? (Probe: What happened in these facilities that made NQAS possible?) - Could you tell us 3-4 suggestions that you have for other facilities that have not yet begun the NQAS certification processes. |
| Challenges | - Could you tell us some of the challenges you face at present with NQAS - Could you tell us a little about the sustainability of the improvements made, after certification? - Could you please explain how you dear with challenges related to finances, HR or other key aspects. Others? |
