# Supplementary material for: What enabled the successful implementation of a quality certification initiative in Bhavnagar, Gujarat? A policy analysis case study
Source: PLOS Glob Public Health. 2025 Jan 21;5(1):e0004180. doi: 10.1371/journal.pgph.0004180 (PMC11750092; doi:10.1371/journal.pgph.0004180)
Supplement: Attachment — Submitted filename: Response to reviewers.docx [file pgph.0004180.s002.docx]

Dear Editors,

We thank you for the peer-review of this article. Your suggestions as well as the reviewers’ comments were very useful in revising the paper. We took some time in revising this since we had to consult with our large group of authors before submission.

In the revised version, we have added depth to some sections and re-arranged some parts to improve readability. A figure is now added to describe the policy triangle framework in the methodology section, in addition to the findings section as suggested by the reviewers. Figure numbers have changed due to this addition, and we have re-submitted the figures with change in the title-numbering. References have also been edited and listed as per the revised content.

We hope that this makes for better reading and improves the paper.

We look forward to hearing from you.

**Point by point response to the editor’s comments:**

Journal Requirements: We would like to request copy editing.

Please review your reference list to ensure that it is complete and correct. If you have cited papers that have been retracted, please include the rationale for doing so in the manuscript text or remove these references and replace them with relevant current references. Any changes to the reference list should be mentioned in the rebuttal letter that accompanies your revised manuscript. If you need to cite a retracted article, indicate the article’s retracted status in the References list and also include a citation and full reference for the retraction notice.
Response: We have reviewed and edited the references.

Additional Editor Comments (if provided):
1. This is an important empirical study, and the paper is overall well-written.

Thank you

2. While the positive model provides valuable insights, it’s equally important to analyse failed models to understand what didn’t work. Authors may explain rationale in studying only positive model.

We agree that lessons can be learnt from both failures and successes. We have now tried to add a stronger justification in the paper for why we chose a positive deviance approach. Positive deviance approaches are often used in examining program implementation and in evaluation, to understand factors underlying success of interventions. Such approaches, in addition to the understanding of barriers, enable the design of future plans and actions; and allow for replication of successful interventions in other contexts. In the case of this study on NQAS, reports on the barriers to certification have been compiled previously (see reference number 10,11, and 12). But positive models have not been documented so far.

3. Please clarify the sampling method used for selecting the five PHFs and specify whether they were from urban, rural, or mixed settings

Details have been added in the methodology section: The five primary healthcare facilities were sampled purposively that had acquired certification in last one year. All facilities were from rural settings.

4. In Table 3B, facilities 1-5 are mentioned, but later it states that four PHFs were selected. Please correct or clarify this inconsistency.

Thank you for pointing out the discrepancy. It has been corrected to five PHFs in the description.

5. In line 195 of the analysis section, there is some repetition regarding the frameworks mentioned. Kindly eliminate the repetition references.

We have eliminated the repetitions and re-arranged the content.

6. The initial part of the discussion (lines 440-452) would be more appropriate in the introduction, particularly in the literature review or rationale section. The authors might consider relocating this content.

Thank you for providing this useful suggestion. We have rearranged the content to include initial parts of the discussion in the introduction section.

7.In line 528, please clarify the different components of the triangle—actors, processes, and context—while removing the repeated word "context."

Thank you for pointing this out. We have corrected the names of components, and brief clarifications have been added against each component.

8. It would be beneficial to shorten the discussion section and include a concise conclusion to enhance readability of the manuscript.

As suggested, we have shortened the discussion section (point no. 7) and have added a conclusions section with concise and clear suggestions based on the study findings.

**Point by point response to the reviewers' comments:**

**Reviewer #1:**

Discussion section:

1. The discussion effectively draws from the findings, but you may want to emphasize the broader implications for other districts or regions in India. A few more explicit recommendations could be added in this section to strengthen the conclusions.

Thank you for this useful suggestion. We have now added the conclusion section with more concise and clear suggestions based on the study findings. These recommendations have broader implications for other districts in India.

2. Since the study uses the "policy triangle framework," you might want to summarize this framework earlier in the introduction to familiarize readers with it before the results section.
The framework has been described in the methodology section and a figure of the policy triangle framework is now added.

Conclusion:
The conclusion is clear but can be enhanced by offering more concrete suggestions for scaling the findings across other districts in India or similar health settings globally.

Included in the conclusion section.

**Reviewer #2:**

Congratulations on being able to conduct an important study.

Thank you.

1. Here are my thoughts and queries:
“In this study, we have used the policy triangle to deepen our understanding of the factors that influenced the implementation of NQAS in Bhavnagar” – need better justification of why the policy triangle framework was used. Why not a framework like “theory of change” that suits the context better?

We agree that several frameworks can be applied to look at the range of success factors in programs. Our early findings suggested that the NQAS certification process was highly political and actor centric. Hence using a framework that explicitly enabled us to capture actors and their interaction with the political context made sense for this study. We have provided a justification for application of the policy triangle as the analytical framework for this study in lines 111-117 (page 5-6).

2. What is done if some parameters remain unmet, and how is compliance ensured/maintained beyond the audit? It should have been included in questions/ “topics” covered in data collection to both district-level actors and facility staff. What is being done to ensure a sustained change (in terms of processes, behaviour, competence) in the way of providing care (clearly hiring ad hoc staff, or getting donations from some community members isn’t sustainable)?

- The study objective was to understand the perspectives of stakeholders on NQAS process within the prescribed framework and we did not include questions on compliance beyond the audit. This has been added in the study limitations.
- Details and a quote on perception of staff on sustainability added in section 2B

3. One of the PHC staff said that they have been able to improve inventory management, prevent stockouts etc. How exactly was the staff member able to do that? How did a checklist-based approach helped the staff member in improve the process?

- We have added some details to the findings section to clarify these points. ‘The checklist-based approach of NQAS process was reported to introduce standardization in inventory management, reducing errors and enabling communication between staff.’
- We did not collect data to verify some of the benefits of NQAS reported by the staff- such as improved service utilization and facility management. This was a qualitative study based on staff perspectives. However, we have added our inability to verify the benefits as a limitation of the study.

4. “Throughout India, the staff is reluctant to participate in NQAS- but that is because the district level teams are not sensitizing them to the importance of NQAS.” What approach did the Bhavnagar team took in sensitizing the staff?

Some of the key lessons from the study findings point to the Bhavnagar district team’s approach in sensitizing the staff, such as- policy champions who motivated the facility staff, and management teams, strong mid-level implementation teams for regular handholding and peer mentors to sensitize the staff on benefits of NQAS certification. These have been described in the discussion and conclusion sections.

5. Study shows that a sense of achievement, as well some healthy competition (for monetary and non-monetary reasons) motivates the staff. But is that enough? Authors could have also asked if there is any deeper meaning that drives them to develop policy framing of NQAS. I would be inclined to think the understanding that “quality matters because people’s well being, and rights matter” could really drive meaningful change by inculcating these values that could be imparted to health providers (beyond a certification process)

Thank you for bringing this important point. We have now strengthened the discussion section to reflect more on this aspect.
