# Supplementary material for: What enabled the successful implementation of a quality certification initiative in Bhavnagar, Gujarat? A policy analysis case study
Source: PLOS Glob Public Health. 2025 Jan 21;5(1):e0004180. doi: 10.1371/journal.pgph.0004180 (PMC11750092; doi:10.1371/journal.pgph.0004180)
Supplement: Attachment — Submitted filename: Response_to_reviewers_auresp_2.docx [file pgph.0004180.s003.docx]

Dear Editors,

We thank you for further suggestions.

In the revised version, we have incorporated the changes suggested in two statements by the editors. The figure files have been reviewed and re-submitted as per the prescribed guidelines of the journal. We have checked the references and ensured that none of the cited papers have been retracted.

We hope that this makes for better reading and improves the paper.

We look forward to hearing from you.

**Point by point response to the editor’s comments:**

A) Journal Requirements:

Please review your reference list to ensure that it is complete and correct. If you have cited papers that have been retracted, please include the rationale for doing so in the manuscript text or remove these references and replace them with relevant current references. Any changes to the reference list should be mentioned in the rebuttal letter that accompanies your revised manuscript. If you need to cite a retracted article, indicate the article’s retracted status in the References list and also include a citation and full reference for the retraction notice.
Response: We have reviewed the references.

B) Additional Editor Comments:

Kindly make below mentioned minor corrections-

1. line 172- check and correct table 3 B title

Response: We have added the description ‘Participant details’ to clarify the title in table 3B as well as table 3A.

2. line 189- remove full form of FGD. You have already given it.

Response: Thank you. We have removed the full form in the given statement.

C) While revising your submission, please upload your figure files to the Preflight Analysis and Conversion Engine (PACE) digital diagnostic tool, <https://pacev2.apexcovantage.com/>. PACE helps ensure that figures meet PLOS requirements. To use PACE, you must first register as a user. Registration is free. Then, login and navigate to the UPLOAD tab, where you will find detailed instructions on how to use the tool. If you encounter any issues or have any questions when using PACE, please email PLOS at figures@plos.org. Please note that Supporting Information files do not need this step.

Response: All the file images have been checked using the PACE tool and re-submitted.
